# The Pepper MAP Kinase CaAIMK1 Positively Regulates ABA and Drought Stress Responses

**DOI:** 10.3389/fpls.2020.00720

**Published:** 2020-05-26

**Authors:** Soongon Jeong, Chae Woo Lim, Sung Chul Lee

**Affiliations:** Department of Life Science (BK21 program), Chung-Ang University, Seoul, South Korea

**Keywords:** ABA, drought, kinase, phosphorylation, stomata

## Abstract

Protein phosphorylation and dephosphorylation are important mechanisms that regulate many cellular processes. Protein kinases usually function in the regulation of the stress responses by adjusting activity via phosphorylation of target proteins. Here, we isolated *CaAIMK1* (*Capsicum annuum* ABA Induced MAP Kinase 1) from the pepper leaves that had been subjected to drought stress. *CaAIMK1* transcripts were induced by drought, abscisic acid (ABA), high salinity, and H_2_O_2_; further, the CaAIMK1-Green fluorescent protein localized in the nucleus and cytoplasm. We performed genetic studies using *CaAIMK1*-silenced pepper plants and *CaAIMK1-*overexpressing (OX) Arabidopsis plants. *CaAIMK1-*silenced pepper plants showed a drought-sensitive phenotype characterized by altered ABA signaling, including low leaf temperatures, and large stomatal apertures. *CaAIMK1-*OX plants exhibited a contrasting drought-tolerant phenotype characterized by decreased levels of transpirational water loss and increased expression levels of Arabidopsis stress-related genes. In *CaAIMK1*^K32N^-OX transgenic Arabidopsis plants, sensitivity to ABA and drought was restored. Collectively, these results demonstrate that CaAIMK1 positively regulates the drought stress responses via an ABA-dependent pathway.

## Introduction

Plants are sessile organisms; therefore, they must adapt to environmental stresses such as pathogen invasion, extreme temperatures, high light intensity, high salinity, and drought stress. Under stress conditions, plants respond via various defense mechanisms, including restriction of growth and development through molecular and physiological changes. Osmotic stress is a critical factor influencing plant growth, development, and survival. The signal transduction pathway underlying osmotic stress responses is dependent on stress-related gene expression, post-translational modification, and hormonal balance (Zhu, 2002; Lim et al., 2015).

The phytohormone abscisic acid (ABA) functions in response to osmotic stress (Cutler et al., 2010; Kim et al., 2010). When plants encounter osmotic stress, ABA is synthesized in several tissues and accumulates in the leaf tissue; this triggers the ABA signal transduction pathway involved in plant adaptive responses (Lee and Luan, 2012; Lim et al., 2015). Abscisic acid signaling is initiated through ABA perception by ABA receptors [the regulatory component of the ABA receptor (RCAR)/pyrabactin resistance 1 (PYR1)/PYR1-like (PYL)] (Lee and Luan, 2012; Lim et al., 2015). After ABA perception, the reversible processes of protein phosphorylation and dephosphorylation occur via sucrose non-fermenting 1 (SNF1)-related protein kinases 2 (SnRK2s) and 2C type protein phosphatases (PP2Cs; Nakashima et al., 2009; Umezawa et al., 2010, 2013). The interaction between clade A PP2Cs and RCARs/PYR1/PYLs inhibits phosphatase activity and releases SnRK2s, leading to the activation of ABA signaling (Lee and Luan, 2012; Lim et al., 2015).

Protein phosphorylation via kinases is the most common post-translational modification process. In eukaryotic cells, 30% of proteins are phosphorylated and 5% of the plant genome encodes kinases (Arabidopsis Genome Initiative, 2000; International Rice Genome Sequencing Project, 2005); 10% of these kinases are associated with mitogen-activated protein kinase (MAPK) cascades. Mitogen-activated protein kinase pathways are composed of three kinases (MAPK, MAPKK, and MAPKKK), which are activated by sequential phosphorylation (de Zelicourt et al., 2016), leading to the induction of transcription factors and several enzymes in response to various biotic and abiotic stresses (Danquah et al., 2015; Mitula et al., 2015). The Arabidopsis genome has 20 MAPKs, 10 MAPKKs, and 80 MAPKKKs (Tena et al., 2001; Xu and Zhang, 2015; Jagodzik et al., 2018); of these, MPK3, MPK4, and MPK6 are the most extensively studied in response to various abiotic stresses and ABA signaling (Ichimura et al., 2000; Xing et al., 2008; Brock et al., 2010; Wu et al., 2015). Previous studies with monocot and dicot plants have suggested that MAPK cascades are involved in ABA signaling processes such as regulation of stomatal apertures, seed germination, and antioxidant defense (Liu, 2012; Danquah et al., 2014; Liu et al., 2015). Under stress conditions, ABA regulates the kinase activity of various MAPK signaling components (Liu, 2012; Danquah et al., 2014). Mitogen-activated protein kinase activity is inhibited and regulated by PP2C in developmental processes and stress signaling pathways. For example, MAPKKK17/18/20 function in ABA responses and is regulated by clade A PP2C ABI1 (Mitula et al., 2015). The MAPKKK17/18-MAPKK3-MAPK1/2/7/14 cascade affects the ABA response—including regulation of stomatal apertures—and the number of stomata, thereby conferring plants with drought tolerance (Danquah et al., 2015; Matsuoka et al., 2015; Mitula et al., 2015).

In the present study, we used RNA-seq analysis to isolate a pepper mitogen-activated protein kinase kinase kinase (MAP3K), *CaAIMK1* (*Capsicum annuum* ABA Induced MAP Kinase 1), from the leaves of pepper plants that had been subjected to ABA treatment (Lim and Lee, 2016). We performed genetic studies using *CaAIMK1*-silenced pepper plants and *CaAIMK1*-overexpressing (OX) transgenic Arabidopsis plants to verify the functional roles of *CaAIMK1* in response to exogenous ABA and drought stress treatments. *CaAIMK1*-silenced pepper plants showed reduced drought tolerance and impaired ABA-mediated stomatal closure. In contrast, *CaAIMK1*-OX plants exhibited ABA hypersensitivity and enhanced drought tolerance. These results suggest that CaAIMK1 functions as a positive regulator of ABA signaling and the drought response.

## Results

### Isolation and Sequence Analysis of the *CaAIMK1* Gene

**FIGURE 1:**
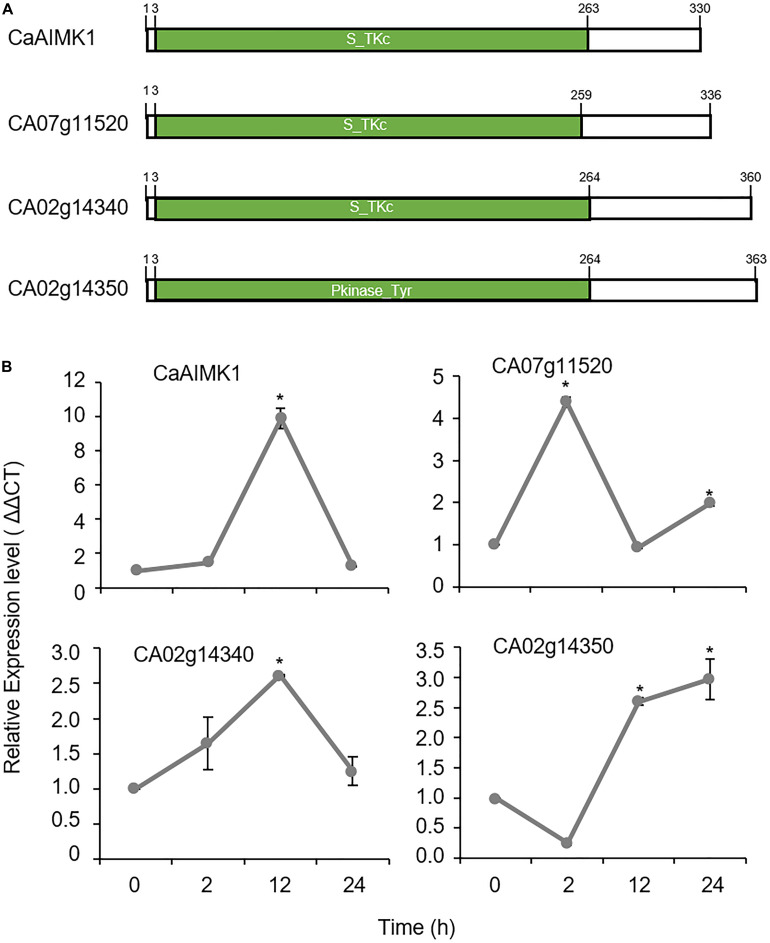
ABA-responsive pepper MAP kinase kinase kinase (MAP3K) genes. **(A)** Domain organization of deduced amino acids in pepper MAP3Ks. The conserved domain was detected using the SMART website (http://smart.embl-heidelberg.de/). **(B)** Expression patterns of *MAP3K*s in pepper leaves in response to ABA treatment. The pepper *Actin1* (*CaACT1*) gene was used as an internal control. Expression level of each gene at 0 h was set to 1.0. Data represent the mean ± standard error (SE) of three independent experiments; asterisks indicate significant difference compared with untreated control (0 h) (Student’s test; *P* < 0.05).

Using RNA-seq analysis, we isolated four genes from the leaves of pepper plants that had been subjected to ABA treatment. We used the webtool SMART^1^ for domain analysis and revealed that these genes have serine-threonine kinase domain or tyrosine kinase domain (Figure 1A). Among them, *CaAIMK1* have the highest fold change and was selected for further analysis (Figure 1B). *CaAIMK1* consists of a 1020-bp open reading frame, encoding 339 amino acid residues with a molecular weight of 38.01 kDa and an isoelectric point of 5.72. Multiple sequence alignment analysis and a protein BLAST search revealed high amino acid sequence identity (36.1–61.5%) between CaAIMK1 and proteins from other plant species; all these proteins contain a highly conserved serine/threonine kinase domain, including VAVK, HCDXXXXN, and DFG motifs (Hanks et al., 1988; Supplementary Figure 1A). Consistently, phylogenetic analysis revealed that CaAIMK1 is clustered with putative pepper MAP kinase and MAP3K kinase kinase kinase proteins from *Arabidopsis thaliana* (Supplementary Figure 1B).

### Molecular Characterization of the CaAIMK1 Protein

**FIGURE 2:**
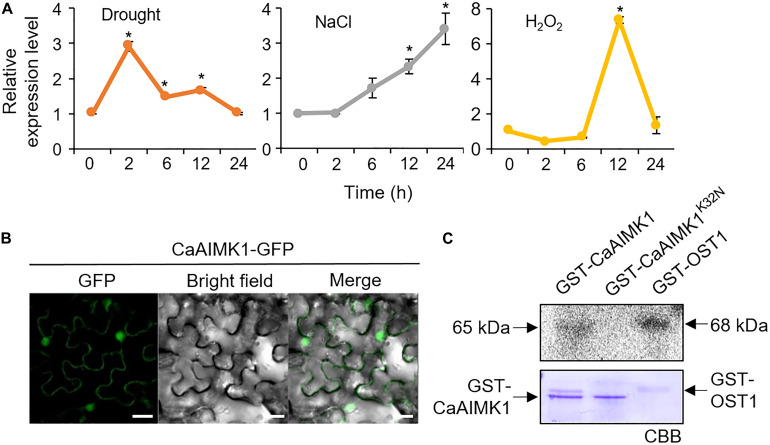
Molecular characterization of CaAIMK1. **(A)** The expression patterns of *CaAIMK1* were examined in the leaves of pepper plants after treatment with 100 μM abscisic acid (ABA), drought, 200 mM NaCl, and 100 μM H_2_O_2_. The pepper *Actin1* gene was used as an internal control. Data represent the mean ± standard error of three independent biological replicates. Asterisks indicate significant differences between 0 h and other times (Student’s *t*-test; *P* < 0.05). **(B)** Subcellular localization of the CaAIMK1-GFP fusion protein in *Nicotiana benthamiana* epidermal cells. The 35S:*CaAIMK1*-*GFP* construct was expressed using agroinfiltration of *N. benthamiana* leaves and observed under a confocal laser−scanning microscope. White bar = 50 μm. **(C)** Auto kinase activity of GST-CaAIMK1 and GST-CaAIMK1^K32N^ using [γ-^32^P]-ATP. GST-OST1 was used as a positive control. The arrows indicates GST-CaAIMK1, GST-CaAIMK1^K32N^, and GST-OST proteins.

To examine the effect of abiotic stresses on *CaAIMK1* expression, we performed quantitative RT-PCR (qRT-PCR) analysis using six-leaf-stage pepper plants (Figure 2A). In drought-treated pepper plants, *CaAIMK1* transcripts were induced 2 h after treatment and subsequently repressed. After exposure to NaCl, *CaAIMK1* expression reached a maximum level 24 h after treatment. The expression level of *CaAIMK1* was also regulated by H_2_O_2_. These data suggest that CaAIMK1 functions in ABA signaling and the abiotic stress responses.

We performed subcellular localization of CaAIMK1 with CaAIMK1-Green fluorescent protein (GFP) fusion protein. Green fluorescent protein signals were strongly detected in the nucleus and weakly detected in the cytoplasm (Figure 2B). To verify the kinase activity of CaAIMK1, we performed an in vitro kinase activity assay by expressing GST-CaAIMK1 and GST-CaAIMK1^K32N^—containing a mutated ATP-binding motif in which Lys-32 is substituted for Asn (Carrera et al., 1993) in *Escherichia coli.* We detected auto kinase activity in GST-CaAIMK1 but not in GST-CaAIMK1^K32N^ (Figure 2C).

### Reduced Drought Tolerance and Decreased ABA Sensitivity in *CaAIMK1*-Silenced Pepper Plants

**FIGURE 3:**
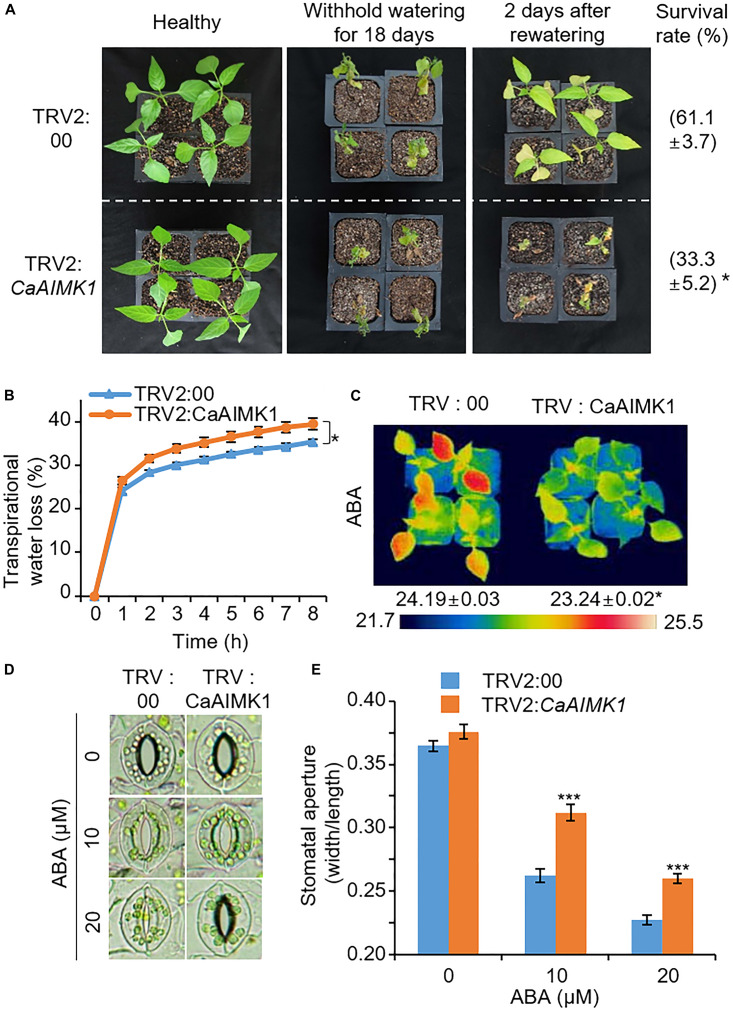
Reduced tolerance of *CaAIMK1*-silenced pepper plants to drought stress. **(A)** Drought susceptibility of *CaAIMK1*-silenced pepper plants. Empty vector control and *CaAIMK1*-silenced pepper plants were grown in pots for 3 weeks under well-watered conditions. The plants were subjected to drought stress by withholding watering for 18 days and then re-watering for 2 days. Representative images were taken before (left) and after (middle) drought treatment and after 2 days of re-watering (right). The survival rates were counted after 2 days of re-watering. Data represent the mean ± standard error of three biological replicates, each evaluating 30 plants. **(B)** Transpirational water loss from the leaves of empty vector control and *CaAIMK1*-silenced pepper plants at various time points after leaf detachment. Data represent the mean ± standard error of three biological replicates, each evaluating 40 leaves. **(C)** Decreased leaf temperatures of empty vector control and *CaAIMK1*-silenced pepper plants after abscisic acid (ABA) treatment. Data represent the mean ± standard error of three biological replicates, each evaluating 10 plants. **(D,E)** Stomatal apertures in empty vector control and *CaAIMK1*-silenced pepper plants after ABA treatment. Representative images were taken under a microscope **(D)** and the stomatal apertures were measured **(E)**. Leaf peels were harvested from 3-week-old pepper plants and incubated in stomatal opening solution containing 0, 10, or 20 μM ABA; the stomatal apertures were then measured under a microscope. Data represent the mean ± standard error of three biological replicates, each evaluating 20 plants. Asterisks indicate significant differences between control and *CaAIMK1*-silenced pepper plants (Student’s *t*-test; * *P* < 0.05, *** *P* < 0.001).

**FIGURE 4:**
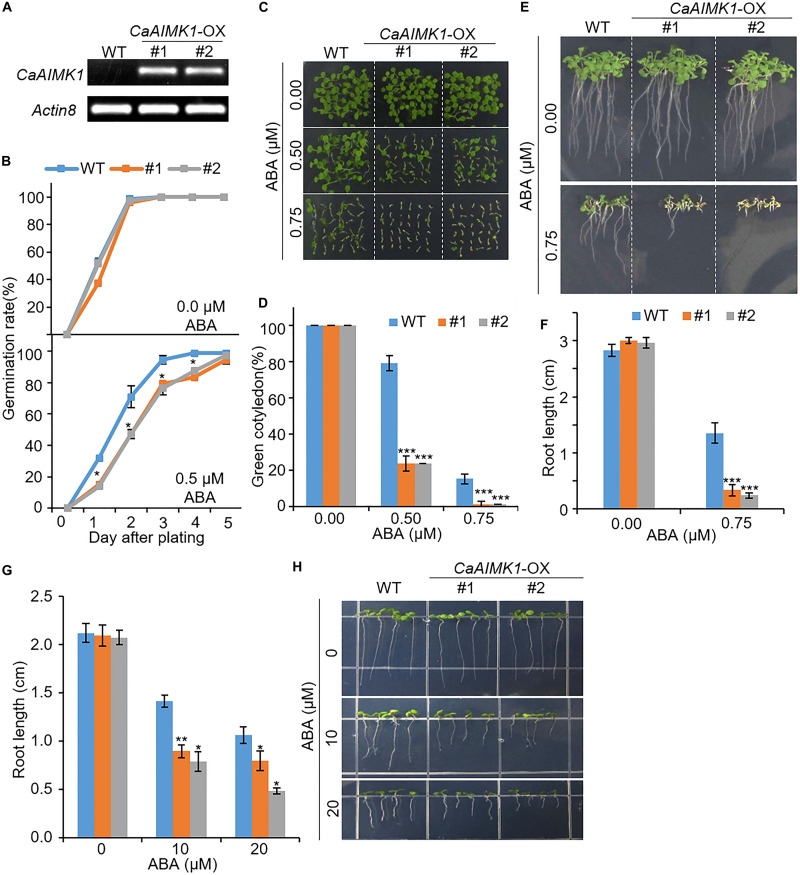
Enhanced sensitivity of *CaAIMK1-*OX transgenic Arabidopsis lines to abscisic acid (ABA). **(A)** Reverse transcription-polymerase chain reaction analysis of *CaAIMK1* expression in wild-type (WT) and *CaAIMK1*-OX transgenic lines. *Actin8* was used as an internal control gene. **(B)** Germination rates of *CaAIMK1-*OX mutants and WT plants on 0.5× Murashige and Skoog (MS) medium containing 0.0 or 0.5 μM ABA. **(C,D)** Seedling development of WT and *CaAIMK1*-OX plants exposed to ABA. Representative photographs were taken 5 days after plating **(C)**. Quantification of green cotyledons in WT and transgenic plants was performed 5 days after plating **(D)**. Data represent the mean ± standard error values obtained after evaluating 72 seeds from three biological replicates. **(E,F)** Primary root elongation of WT and transgenic lines exposed to ABA. Representative images were taken **(E)**, and the root length of each plant was measured 8 days after sowing **(F)**. **(G,E)** Post germinative growth of WT and transgenic lines. Plants were grown on 0.5× Murashige and Skoog (MS) medium for 2 days and transferred onto 0.5× MS medium containing 0, 10 or 20 μM ABA. After 5 days, root length of WT and transgenic lines were measured **(G)** and representative photographs were taken **(H)**. Data represent the mean ± standard error of three biological replicates, each evaluating 25 seeds. Asterisks indicate significant differences between WT and transgenic lines (Student’s *t*-test; * *P* < 0.05, *** *P* < 0.001).

**FIGURE 5:**
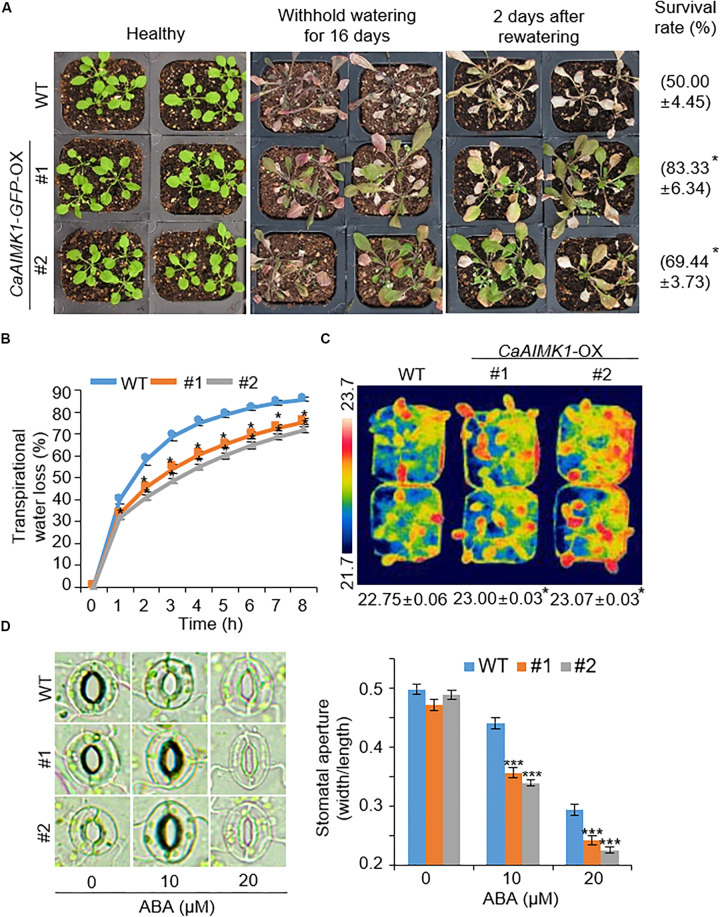
Enhanced tolerance of *CaAIMK1*-OX plants to drought stress. **(A)** Drought-tolerant phenotype of *CaAIMK1*-OX plants. Three-week-old wild-type (WT) and transgenic plants were subjected to drought stress by withholding watering for 16 days and then re-watering for 2 days. Representative images were obtained before (left) and after (middle) drought and after 2 days of re-watering (right). Survival rates of plants after 2 days of re-watering. Data represent the mean ± standard error of three biological replicates, each evaluating 24 plants. **(B)** Transpirational water loss from the leaves of WT and transgenic plants at various time points after leaf detachment. **(C)** Increased leaf temperatures of *CaAIMK1*-OX plants in response to abscisic acid (ABA) treatment. Data represent the mean ± standard error of three biological replicates, each evaluating 10 plants. **(D,E)** Stomatal apertures in WT and *CaAIMK1*-OX plants exposed to ABA. Leaf peels were harvested from 3-week-old plants of each line and incubated in stomatal opening solution containing 0, 10, or 20 μM ABA. Representative images were taken under a microscope **(D)**, and stomatal apertures were measured **(E)**. Data represent the mean ± standard error of three biological replicates, each evaluating 20 plants. Asterisks indicate significant differences between WT and transgenic lines (Student’s *t*-test; * *P* < 0.05, *** *P* < 0.001).

**FIGURE 6:**
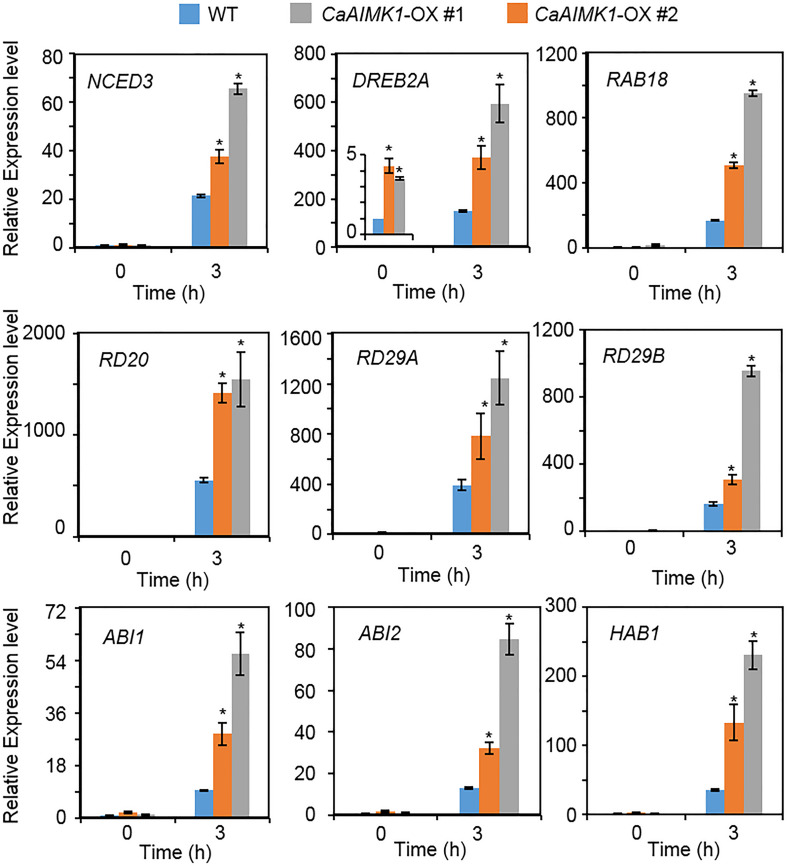
Drought-inducible genes in *CaAIMK1*-OX plants. Quantitative reverse transcription-polymerase chain reaction analysis of *CaAIMK1*-OX plants exposed to drought stress at 3 h after leaf detachment. The relative expression levels (ΔΔCT) of each gene were normalized to the those of *Actin8* as an internal control gene. Data represent the mean ± standard error of three biological replicates. Asterisks indicate significant differences between wild-type (WT) and transgenic lines (Student’s *t*-test; * *P* < 0.05).

Pepper plants have very low transformation efficiency; hence, we performed genetic analysis of CaAIMK1 using virus-induced gene silencing (VIGS) and overexpression in pepper plants and Arabidopsis plants, respectively (Figures 3–6). The expression level of CaAIMK1 is low in *CaAIMK1*-silenced pepper compared to control plants (Supplementary Figure 2). To examine the effect of *CaAIMK1* silencing on drought response, we subjected *CaAIMK1*-silenced and control pepper plants to drought stress by withholding watering for 18 days and then re-watering for 2 days. Under these conditions, *CaAIMK1*-silenced pepper plants exhibited more wilted phenotypes than control plants (Figure 3A, middle and right panels). The survival rates of control and *CaAIMK1*-silenced pepper plants were 61.1 and 33.3%, respectively. The water retention capacity conferred by the inhibition of transpirational water loss is critical for the determination of drought tolerance; hence, we examined the water retention capacity by measuring leaf fresh weight (Figure 3B). The fresh weight loss was significantly higher in *CaAIMK1*-silenced pepper plants than in control pepper plants from 1 to 8 h after detachment. To determine the relationship between drought stress responses and ABA-dependent stomatal closure, we measured leaf temperatures and stomatal apertures in *CaAIMK1*-silenced and control pepper plants (Figures 3C–E). The leaf temperatures were significantly lower in *CaAIMK1*-silenced pepper plants than in control pepper plants (Figure 3C). In plants, evaporative cooling is derived from stomatal closure, which leads to elevated leaf temperatures (Schroeder et al., 2001); hence, we postulated that lower leaf temperatures in *CaAIMK1*-silenced pepper plants were derived from enhanced stomatal opening. We measured stomatal apertures in *CaAIMK1*-silenced pepper plants and control pepper plants (Figures 3D,E). In the absence of ABA, almost all the stomata in the leaves of *CaAIMK1*-silenced pepper and control plants were open. After treatment with 10 and 20 μM ABA, *CaAIMK1*-silenced pepper and control plants displayed stomatal closure; however, the stomatal apertures were significantly larger in *CaAIMK1*-silenced pepper plants than in control plants. Collectively, these results demonstrate that CaAIMK1 functions in response to drought stress via ABA-dependent signaling.

### Enhanced ABA Sensitivity in *CaAIMK1*-OX Transgenic Arabidopsis Plants

For further genetic analysis of CaAIMK1, we generated *CaAIMK1*-OX transgenic Arabidopsis plants, which constitutively expressed *CaAIMK1* (Figures 4–6). We selected two independent transgenic lines—*CaAIMK1*-OX #1 and *CaAIMK1*-OX #2—and used these plants in our subsequent phenotypic assays. First, we used semi-quantitative RT-PCR analysis to examine the expression level of *CaAIMK1*. We detected *CaAIMK1* transcripts in *CaAIMK1*-OX plants but not in wild-type plants (Figure 4A). *CaAIMK1* expression was induced by ABA (Figure 1); hence, we investigated the effect of ABA on *CaAIMK1*-OX plants at the germination and seedling stages (Figures 4B–F). In the absence of ABA, the germination rates did not differ significantly between *CaAIMK1*-OX and wild-type plants; however, in the presence of ABA, the germination rates of *CaAIMK1*-OX seeds were significantly lower those of wild-type plants (Figure 4B). Moreover, at the seedling stage, *CaAIMK1*-OX plants displayed ABA-hypersensitive phenotypes characterized by low rates of cotyledon greening and decreased primary root growth (Figures 4C–F). To determine whether the ABA hypersensitivity at seedling stage is caused from the influence of ABA on germination or seedling growth, we have performed post-germination assay with ABA (Figures 4G,H). The primary root growth of *CaAIMK1*-OX plants was significantly longer than that of wild-type plants. These results indicate that enhanced expression of *CaAIMK1* in Arabidopsis confers ABA hypersensitivity during the germination and seedling stages.

### Enhanced Drought Tolerance in *CaAIMK1*-OX Transgenic Arabidopsis Plants

To further elucidate the biological role of CaAIMK1 in response to drought stress, we subjected wild-type and *CaAIMK1*-OX plants to drought stress. After 3 weeks under well-watered conditions, we observed no phenotypic differences between wild-type and *CaAIMK1*-OX plants (Figure 5A, left panel). However, when we subjected plants to drought stress by withholding watering for 16 days and then re-watering for 2 days, *CaAIMK1*-OX plants displayed a less wilted phenotype than wild-type plants (Figure 5A, middle and right panels). Moreover, survival rates of wild-type and transgenic plants were 50.00% and 69.44–83.33%, respectively (Figure 5A). Next, we analyzed the transpirational water loss by measuring the fresh weight of detached rosette leaves (Figure 5B). The fresh weight loss was significantly lower in *CaAIMK1*-OX plants than in wild-type plants from 1 to 8 h after detachment. We investigated ABA sensitivity by measuring leaf temperatures and stomatal apertures (Figures 5C–E). In the presence of ABA, the leaf temperatures of *CaAIMK1*-OX plants were significantly higher than those of wild-type plants (Figure 5C). In the absence of ABA, we determined no significant difference in stomatal apertures between wild-type and *CaAIMK1*-OX plants (Figures 5D,E). However, after treatment with 10 and 20 μM ABA, *CaAIMK1*-OX plants had significantly smaller stomatal pores than wild-type plants. Collectively, these data indicate that *CaAIMK1*-OX plants display an enhanced capacity for water retention derived from ABA hypersensitivity, and this confers a drought-tolerant phenotype.

To verify the molecular mechanism whereby enhanced expression of *CaAIMK1* influences ABA biosynthesis, ABA signaling, and drought signaling, we conducted a qRT-PCR assay using stress-related genes (Figure 6). When plants were grown under well-watered conditions, the expression levels of stress-related genes generally did not differ significantly between wild-type and *CaAIMK1*-OX plants; the exception was *DREB2A*, which was induced significantly more strongly in *CaAIMK1*-OX plants than in wild-type plants. When plants were subjected to drought stress, stress-related gene expression was strongly induced in *CaAIMK1*-OX and wild-type plants after 3 h; moreover, expression levels of all the investigated genes were significantly higher in *CaAIMK1-*OX plants than in wild-type plants. These results indicate that the drought-tolerant phenotype displayed by *CaAIMK1-*OX plants is derived from the modulation of stress-related gene expression.

### Recovery of ABA and Drought Sensitivity in *CaAIMK1*^K32N^-OX Transgenic Arabidopsis Plants

**FIGURE 7:**
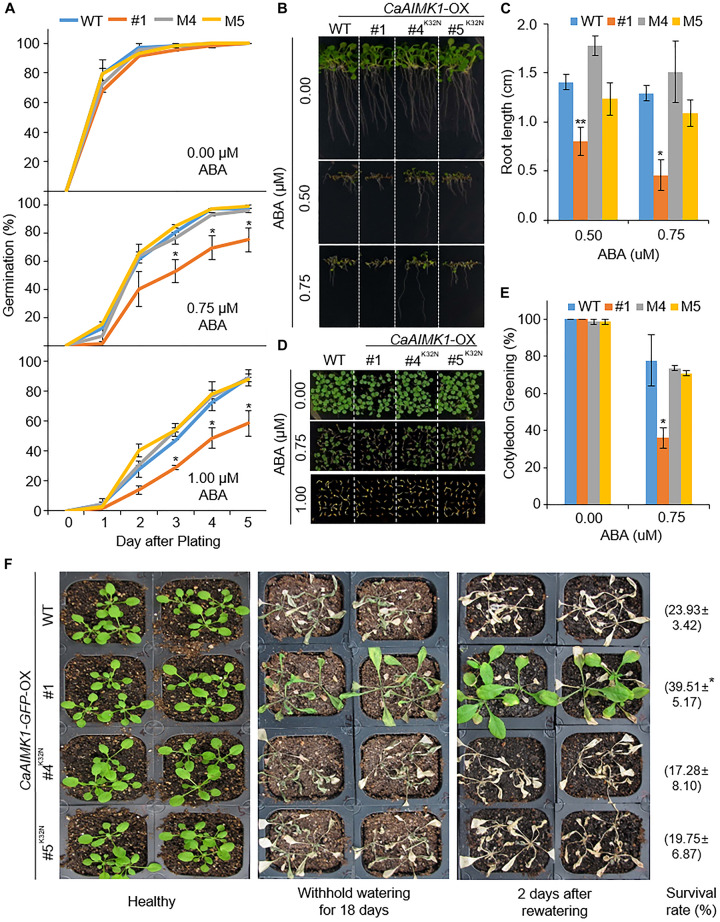
Restored sensitivity of *CaAIMK1*^K32N^-OX plants to abscisic acid (ABA) and drought stress. **(A)** Germination rates of wild-type (WT), *CaAIMK1-*OX mutant, and *CaAIMK1*^K32N^-OX plants on 0.5× Murashige and Skoog (MS) medium containing 0, 0.75, or 1.00 μM ABA. **(B,C)** Primary root elongation of WT and transgenic lines exposed to ABA. Representative images were taken **(B)** and the root length of each plant was measured 8 days after sowing **(C)**. **(D,E)** Seedling development of WT, *CaAIMK1*-OX, and *CaAIMK1*^K32N^-OX plants exposed to various concentrations of ABA. Representative photographs were taken 5 days after plating **(D)**. Quantification of green cotyledons in WT and transgenic plants was performed 5 days after plating **(E)**. Data represent the mean ± standard error values obtained after evaluating 72 seeds from three biological replicates. **(F)** Drought-sensitive phenotype displayed by *CaAIMK1*^K32N^-OX plants. Three-week-old WT and transgenic plants were subjected to drought stress by withholding watering for 18 days and then re-watering for 2 days. Representative images were obtained before (left panel) and after (middle panel) drought stress and after 2 days of re-watering (right panel). Survival rates of plants after 2 days of re-watering are shown in parentheses. Data represent the mean ± standard error of three biological replicates, each evaluating 27 plants. Asterisks indicate significant differences between WT and transgenic lines (Student’s *t*-test; * *P* < 0.05, ** *P* < 0.01).

To verify that the kinase domain of CaAIMK1 is critical for ABA sensitivity and the drought response, we generated *CaAIMK1*^K32N^-OX transgenic Arabidopsis plants—containing a mutated ATP-binding motif in which Lys-32 is substituted for Asn (Carrera et al., 1993). We selected two independent transgenic lines (*CaAIMK1*^K32N^-OX #4 and *CaAIMK1*^K32N^-OX #5) and used these lines in our subsequent phenotypic analysis of response to ABA and drought stress (Figure 7). First, we verified ABA phenotypes of transgenic plants at the germination and seeding stages (Figures 7A–E). In the absence of ABA, we observed no phenotypic differences between wild-type, *CaAIMK1*-OX, and *CaAIMK1*^K32N^-OX plants. However, when plants were exposed to ABA, wild-type and *CaAIMK1*^K32N^-OX plants displayed less-sensitive phenotypes than *CaAIMK1*-OX plants as measured by germination rates (Figure 7A), primary root length (Figures 7B,C), and cotyledon greening rates (Figures 7D,E). We subjected plants to drought stress by withholding watering for 18 days and then re-watering for 2 days. Under well-watered conditions, we observed no phenotypic differences between wild-type, *CaAIMK1*-OX, and *CaAIMK1*^K32N^-OX plants (Figure 7F, left panel). However, after exposure to drought stress, wild-type and *CaAIMK1*^K32N^-OX plants displayed a more wilted phenotype than *CaAIMK1*-OX plants (Figure 7F, middle and right panels). Moreover, the survival rates of *CaAIMK1*^K32N^-OX plants were comparable with those of wild-type plants. These results indicated that the kinase domain of CaAIMK1 is important for ABA signaling and drought stress responses.

## Discussion

To adapt to water-deficit conditions, plants regulate various cellular processes, including transcription, translation, and post-translational modification. Protein phosphorylation via plant kinases is a post-translational modification process, and it plays an important role in ABA signaling and the drought stress responses (Bundo and Coca, 2017; Yang et al., 2017). Several stress-related kinases have been identified and functionally characterized; nevertheless, the precise mechanisms by which plants respond to stress remain unclear. Among stress-related kinases, SnRK2 type kinases (SnRK2s) and receptor-like kinases (RLKs) are the most extensively studied protein kinases involved in the adaptation to drought stress. SnRK2s function in the ABA core signaling pathway and are associated with defense responses, including the regulation of transcription and stomatal apertures (Lee and Luan, 2012; Lim et al., 2015). Receptor-like kinases perceive stress and transport signals to downstream target proteins via phosphorylation (Shiu and Bleecker, 2003; Gish and Clark, 2011). MAPKs are also involved in ABA signaling and stress responses (Danquah et al., 2014); however, they have been less extensively investigated than SnRK2s and RLKs. Recent studies have characterized MAPKs associated with RCAR-PP2C-SnRK2 ABA core signaling; these MAPKs play an important role in plant adaptation to drought stress (Danquah et al., 2015; Mitula et al., 2015). Nevertheless, information is lacking on the involvement of MAPKs—especially pepper plant MAPKs—in the stress responses. In the present study, we identified and functionally characterized the ABA-induced MAP3K gene *CaAIMK1*. We showed that the CaAIMK1 protein positively regulates drought tolerance, mainly by modulating ABA sensitivity.

Among Arabidopsis MAPKs, MAPKKK15, MAPKKK16, MAPKKK17, and MAPKKK18 are closely related to CaAIMK1 with 40.6–37.8% identity and 64.6–52.5% similarity (Supplementary Figure 1B). MAPKKK17 and MAPKKK18 are induced by ABA and osmotic stresses (Danquah et al., 2015). The kinase activity of MAPKKK18 is affected by ABA, and its stability is modulated by ABA core signaling (Mitula et al., 2015). Amino acid alignment enables the detection of highly conserved domains—including VAVK, HCDXXXXN and DFG motifs—in MAPKs (Supplementary Figure 1A). Using an *in vitro* kinase activity assay, we showed that kinase activity is impaired by substitution of Lys-32 for Asn; hence, the Lys residue is essential for kinase activity of CaAIMK1. The substitution of Lys-32 for Asn influenced CaAIMK1 function in ABA sensitivity and drought tolerance, indicating that kinase activity is critical for the ABA-mediated drought response.

In the present study, we further showed that the levels of several stress-related genes associated with ABA biosynthesis, ABA signaling, and stress responses increased under different stress conditions. Altered expression of these genes is critical for the determination of stress sensitivity or stress tolerance (Hubbard et al., 2010; Fujita et al., 2011; Joo et al., 2018; Lim et al., 2018). Several stress-related genes—including *NCED3*, *DREB2A*, *RD29A*, and *PP2C*s—were more strongly expressed in *CaAIMK1*-OX plants than in wild-type plants. Under well-watered conditions, we observed no phenotypic differences or significantly different expression of stress-related genes between wild-type and *CaAIMK1*-OX plants. However, when plants were subjected to drought stress, *CaAIMK1*-OX plants displayed a less wilted phenotype; this was associated with altered water retention capacity, indicating that *CaAIMK1* expression enhances stomatal closure. Induction of *NCED3* is critical for ABA biosynthesis in plants (Iuchi et al., 2001; Tan et al., 2003). Moreover, increased ABA levels trigger stomatal closure in guard cells as the rapid process for inhibiting water loss (Schroeder et al., 2001). Group A PP2Cs function as negative regulators in ABA signaling; however, after ABA treatment or under stress conditions, *PP2C*s are induced. Hence, the enhanced drought tolerance displayed by *CaAIMK1*-OX plants is likely derived from different endogenous ABA contents and altered response to ABA. Our qRT-PCR analysis revealed that *CaAIMK1* modulates the expression of several stress-related genes, including *NCED3*, *DREB2A*, *RD29A*, and *PP2C*s; hence, we propose that, under drought stress conditions, CaAIMK1 functions upstream of these genes. Nevertheless, enhanced expression of stress-related genes does not fully explain the ABA-sensitive and drought-tolerant phenotypes displayed by *CaAIMK1*-OX plants.

In summary, our study provides several lines of evidence that CaAIMK1 functions as a positive regulator of ABA signaling and drought tolerance. Our data indicate that the kinase activity of CaAIMK1 modulates the drought stress responses via the regulation of stomatal closure and stress-related gene expression. However, the downstream MAPK cascade and target proteins that are modulated by CaAIMK1 remain elusive. Further studies to identify the CaAIMK1 downstream MAPK cascade and the target protein that is phosphorylated and regulated by this MAPK cascade are required. Elucidation of the function of the ABA-activated MAPK cascade and its target protein will provide valuable insights into the complex process of plant stress tolerance and will make an important contribution to sustainable agriculture.

## Materials and Methods

### Plant Materials and Growth Conditions

Pepper (*C. annuum* cv. Nockwang), tobacco (*Nicotiana benthamiana*), and Arabidopsis (ecotype Col-0) plants were used in this study. Seeds were sown in a sterilized compost soil mix (peat moss, perlite, and vermiculite, 5:3:2, v/v/v), sand, and loam soil (1:1:1, v/v/v). The pepper plants were grown in a growth room at 27 ± 1°C under white fluorescence (130 μmol photons m^–2^ s^–1^) with a 16-h light/8-h dark cycle per day. The tobacco plants were grown in a growth room at 25 ± 1°C. *Arabidopsis thaliana* (ecotype Col-0) seeds were vernalized at 4°C for 2 days to synchronize germination and germinated on Murashige and Skoog (MS) medium (Duchefa Biochemie, Haarlem, Netherlands) supplemented with 1% sucrose and 0.8% agarose (Duchefa Biochemie). Arabidopsis seedlings were grown under 130 μmol photons⋅m^–2^ s^–1^ fluorescence in a sterilized compost soil mix (peat moss, perlite, and vermiculite, 9:1:1, v/v/v) at 24°C with 60% relative moisture.

### Generation of *CaAIMK1*-OX Arabidopsis Plants

For the generation of *CaAIMK1*-OX Arabidopsis plants, the full-length coding sequence of *CaAIMK1* was inserted into the pENTR/D−TOPO vector (Invitrogen, Carlsbad, CA, United States). The 35S promoter-driven *CaAIMK1-GFP* construct was generated via the LR reaction. This construct was transformed into *Agrobacterium tumefaciens* strain GV3101 using heat shock methods and then transformed into Arabidopsis via the floral dip method (Clough and Bent, 1998). For the selection of *CaAIMK1*-OX plants, T_3_ seeds were plated on MS agar supplemented with 25 μg ml^–1^ phosphinothricin.

### Virus-Induced Gene Silencing

For the analysis of *CaAIMK1* knockdown pepper plants, the tobacco rattle virus (TRV)-based VIGS system was used to generate loss-of function *CaAIMK1* pepper plants as described by Lim et al. (2017). pTRV1, pTRV2:*CaAIMK1* (1–160 bp), and pTRV2:00 (as a negative control) were transformed into *A. tumefaciens* strain GV3101 and then infiltrated into cotyledons of pepper plants (OD_600_ = 0.2 for each construct).

### Subcellular Localization Analysis

Green fluorescent protein-tagged *CaAIMK1* was agroinfiltrated into 5-week-old *N. benthamiana* leaves for transient expression. *A. tumefaciens* strain GV3101 carrying the gene construct was combined with p19 (each construct, OD_600_ = 0.5, 1:1 ratio). At 2 days after agroinfiltration, the GFP signals were detected using a confocal microscope (510 UV/Vis Meta; Zeiss).

### *In vitro* Kinase Activity Assay

For the *in vitro* kinase activity assay, we used kinase buffer, which was composed of 1 mM CaCl_2_, 2.5 mM MgCl_2_, 2.5 mM MnCl_2_, 20 mM Tris-HCl (pH 7.5), and 1 mM dithiothreitol. The total volume of 40 μL included the protein mixture with 7.5 μCi [γ-^32^P]-ATP. After reaction at 30°C for 3 h, the reaction was stopped by adding 10 μL of 5× SDS sample buffer containing 250 mM Tris-HCl (pH 6.8), 10% SDS, 50% glycerol, 0.005% bromophenol blue (G-250), and 25% β-mercaptoethanol; 15 μL of the mixture was then loaded onto a 10% SDS-PAGE gel. The gel was dried using a gel dryer (Bio-Rad), and ^32^P was detected through autoradiography using a Personal Molecular Imager (Bio-Rad).

### ABA, Dehydration, NaCl, and H_2_O_2_ Treatments

To examine the *CaAIMK1* expression level in pepper plants, we used six-leaf-stage pepper plants. We sprayed plants with ABA (100 μM) or H_2_O_2_ (100 μM) or irrigated plants with NaCl (200 mM). Leaves were harvested at 0, 2, 6, 12, and 24 h with three independent experiments. To determine the expression levels of stress-related genes, we removed 4-week-old wild-type and *CaAIMK1*-OX transgenic plants from soil in order to be subjected to dehydration stress, and the leaves were harvested 0 and 3 h after treatment.

### RNA Precipitation and Real Time–Reverse Transcription-Polymerase Chain Rreaction

Total RNA was precipitated from pepper and Arabidopsis leaves. All samples were prepared with RNase and DNase free water, and genomic DNA was removed using RNA free-DNase (Qiagen, Valencia, CA, United States). Using a spectrophotometer, total RNA was quantitated to 1 μg for a template to synthesize cDNA using an iScript^TM^ cDNA synthesis kit (Bio-Rad). The cDNA was amplified using iQ^TM^SYBR Green Supermix with specific primers (Supplementary Table 1) and a CFX96 Touch^TM^ Real−Time PCR detection system (Bio−Rad, Hercules, CA, United States). Pepper *Actin1* (*CaACT1*) and Arabidopsis *Actin8* (*AtACT8*) genes were used as standard controls.

### Phenotypic Assays

Four-leaf-stage pepper plants and 3-week-old Arabidopsis plants were grown under well-watered conditions in a growth room. Water was withheld for 16 and 18 days in pepper plants and Arabidopsis plants, and the plants were then re-watered for 2 days. We counted the number of plants that resumed their growth. For the measurement of transpirational water loss, the first and second leaves and six rosette leaves were detached from four-leaf-stage pepper plants and 4-week-old Arabidopsis plants, respectively, and placed in a Petri dish. The detached leaves were maintained at 40% relative humidity, and the fresh weights were measured at the indicated time points.

To measure the leaf temperature, pepper and Arabidopsis plants were sprayed with 100 and 50 μM ABA, respectively. Leaf temperature photographs were taken using an infrared camera (T420; FLIR systems) and gauged with ver. 5.13 software FLIR Tools+.

For the measurement of stomatal pore size, pepper and Arabidopsis leaves were harvested. Leaves were floated on stomatal opening solution (50 mM KCl, 10 mM MES-KOH, and 10 μM CaCl_2_, pH 6.15) for 3 h and then transferred to stomatal opening solution with or without ABA (10 and 20 μM) for 2.5 h at 24°C. After incubation, each stomata sample was randomly sampled and photographed under a Nikon Eclipse 80i microscope.

## Data Availability Statement

The raw data supporting the conclusions of this article will be made available by the authors, without undue reservation, to any qualified researcher.

## Author Contributions

SJ and CL performed the experiments and analyzed the results. SL designed the experiments and wrote the manuscript.

## Conflict of Interest

The authors declare that the research was conducted in the absence of any commercial or financial relationships that could be construed as a potential conflict of interest.

## Footnotes

1 http://smart.embl-heidelberg.de/
