# Supplementary material for: The Pepper MAP Kinase CaAIMK1 Positively Regulates ABA and Drought Stress Responses
Source: Front Plant Sci. 2020 May 26;11:720. doi: 10.3389/fpls.2020.00720 (PMC7264397; doi:10.3389/fpls.2020.00720)
Supplement: Supplementary file 1 [file Image_1.PDF]

A

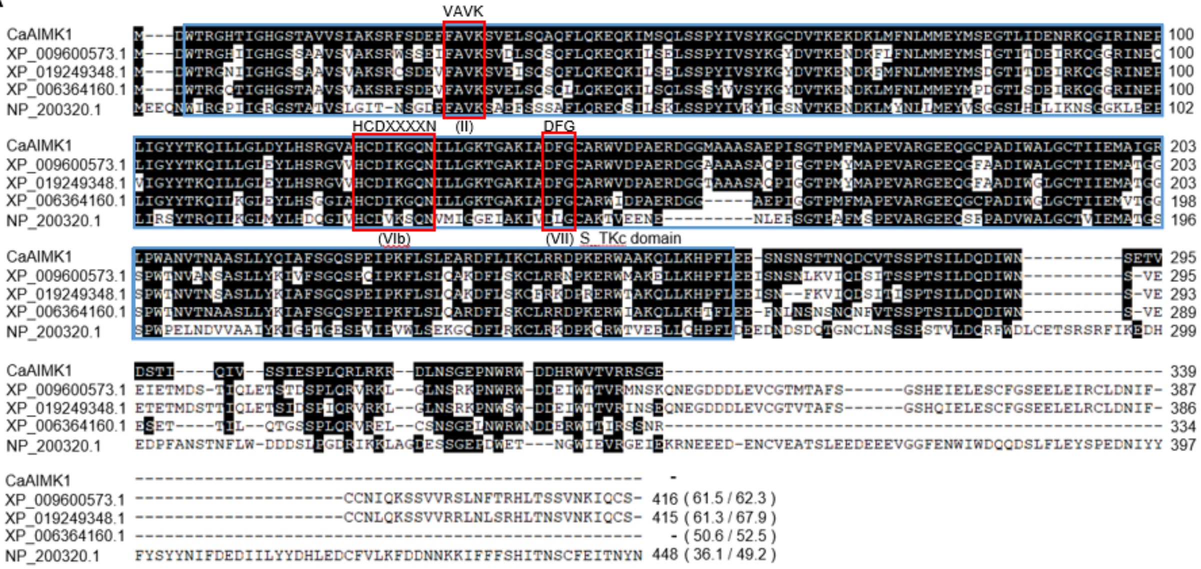
VAVK
HCDXXXXN
DFG
(II)
(VIb)
(VII)
S_TKc domain
CaAIMK1
XP_009600573.1
XP_019249348.1
XP_006364160.1
NP_200320.1
416 (61.5 / 62.3)
415 (61.3 / 67.9)
- (50.6 / 52.5)
448 (36.1 / 49.2)

B

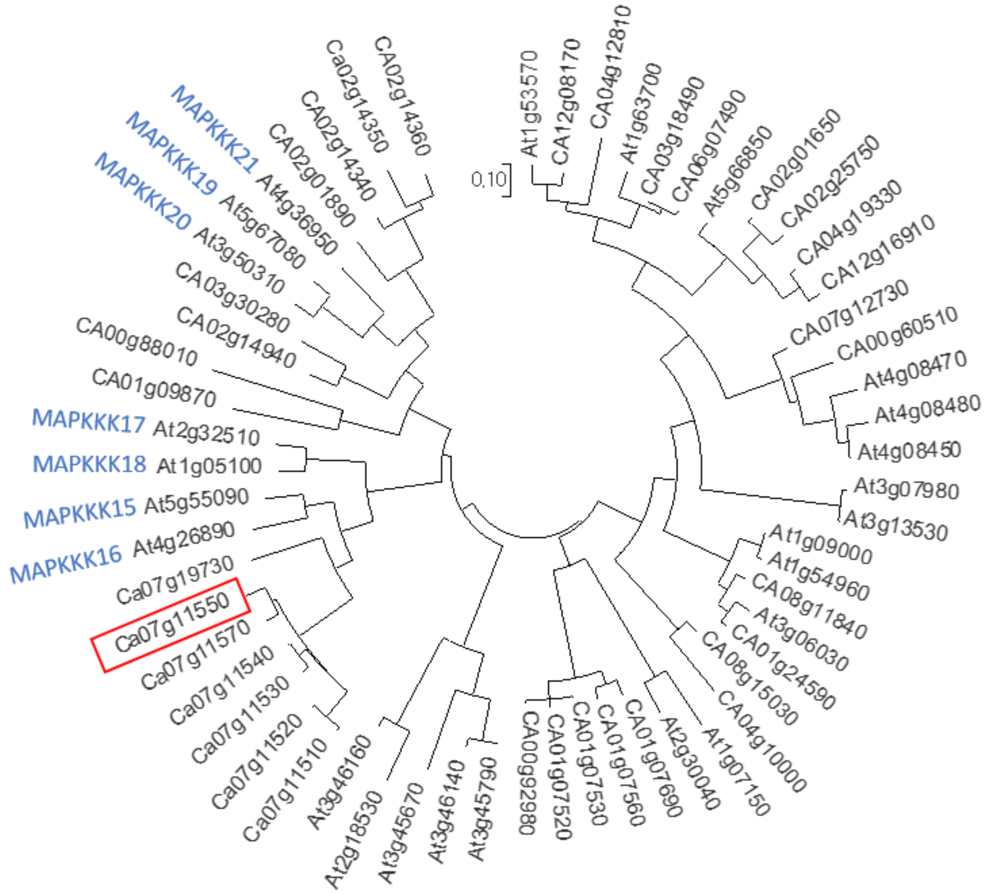
0.10
MAPKKK21 At4g36950
MAPKKK19 At5g67080
MAPKKK20 At3g50310
MAPKKK17 At2g32510
MAPKKK18 At1g05100
MAPKKK15 At5g55090
MAPKKK16 At4g26890
Ca02g14350
CA02g14360
CA02g14340
CA02g01890
CA03g30280
CA02g14940
CA00g88010
CA01g09870
Ca07g19730
Ca07g11550
Ca07g11570
Ca07g11540
Ca07g11530
Ca07g11520
Ca07g11510
At3g46160
At2g18530
At3g45670
At3g46140
At3g45790
CA00g92980
CA01g07520
CA01g07530
CA01g07560
CA01g07690
At2g30040
At1g07150
CA04g10000
CA08g15030
CA01g24590
At3g06030
CA08g11840
At1g54960
At1g09000
At3g13530
At3g07980
At4g08450
At4g08480
At4g08470
CA00g60510
CA07g12730
CA12g16910
CA04g19330
CA02g25750
CA02g01650
At5g66850
CA06g07490
CA03g18490
At1g63700
CA04g12810
CA12g08170
At1g53570

Supplementary Figure 1. Amino acid sequence analysis of pepper CaAIMK1. (A) Comparisons of the deduced amino acid sequences of the CaAIMK1 protein with those of *Nicotiana tomentosiformis* (accession no. XP_009600573.1), *Nicotiana attenuate* (accession no. XP_019249348.1), *Solanum tuberosum* (accession no. XP_006364160.1), and *Arabidopsis thaliana* (accession no. NP_200320.1) proteins. Identical amino acid residues are highlighted in black. Blue box indicates kinase domain; red boxes indicate VAVK, HRDXXXXN, and DFG motifs. (B) Molecular phylogenetic analysis of the CaAIMK1 protein. Phylogenetic analysis was conducted using MEGA software (version 7.0) with its homologous Arabidopsis and pepper proteins. Red box indicates the CaAIMK1 protein.
