# Supplementary material for: The Pepper MAP Kinase CaAIMK1 Positively Regulates ABA and Drought Stress Responses
Source: Front Plant Sci. 2020 May 26;11:720. doi: 10.3389/fpls.2020.00720 (PMC7264397; doi:10.3389/fpls.2020.00720)
Supplement: Supplementary file 2 [file Image_2.PDF]

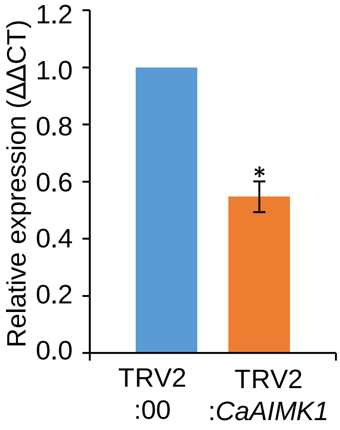

Supplementary Figure 2 qRT-PCR analysis of *CaAIMK1* gene expression in the leaves of *CaAIMK1*-silenced pepper plants (TRV:*CaAIMK1*) and empty vector control pepper plants (TRV:00) 12 h after treatment with 100 µM ABA. The relative expression levels (ΔΔCT) of each gene were normalised to the geometric mean of *Actin8* as an internal control gene. Data represent the mean ± standard error of three biological replicates. Asterisks indicate significant differences between wild-type (WT) and transgenic lines (Student's *t*-test; *$P < 0.05$).
