# Supplementary material for: The Pepper MAP Kinase CaAIMK1 Positively Regulates ABA and Drought Stress Responses
Source: Front Plant Sci. 2020 May 26;11:720. doi: 10.3389/fpls.2020.00720 (PMC7264397; doi:10.3389/fpls.2020.00720)
Supplement: Supplementary file 3 [file Table_1.PDF]

Supplementary Table 1. Sequences of primers used in this study.

Primer name		Primer sequence (5' - 3')
**For cloning**		
CaAIMK1-CDS	Forward	ATGGATTGGACCAGAGGCCATA
	Reverse	CTATTCACCGCTTCTTCTAACAGTAAC
	Forward (w/o stop codon)	CGAATTCGCCCTTTTCACCGCTTCTTCTAACAGTAAC
	Reverse (w/o stop codon)	GTTACTGTTAGAAGAAGCGGTGAAAAGGGCGAATTCG
CaAIMK1-VIGS	Forward	TCTAGAATGGATTGGACCAGAGGC
	Reverse	CTCGAGAGCTCAATTGCGACATAAT
CaAIMK1 K32N	Forward	GGATAGCTCCACGGAGTTGACAGCAAAAAATTCATCG
	Reverse	CGATGAATTTTTTGCTGTCAACTCCGTGGAGCTATCC
**For RT-PCR**		
CaAIMK1	Forward	ATGGATTGGACCAGAGGCCATAC
	Reverse	TGGCTCATTGATCCGAATACCC
CaACT1	Forward	GACGTGACCTAACTGATAACCTGAT
	Reverse	CTCTCAGCACCAATGGTAATAACTT
NCED3	Forward	ACATGGAAATCGGAGTTACAGATAG
	Reverse	AGAAACAACAAACAAGAAACAGAGC
DREB2A	Forward	CTACAAAGCCTCAACTACGGAATAC
	Reverse	AAACTCGGATAGAGAATCAACAGTC
RAB18	Forward	GGAAGAAGGGAATAACACAAAAGAT
	Reverse	GCGTTACAAACCCTCATTATTTTTA
RD20	Forward	TGGTTTCCTATCTAAAGAAGCTGTG
	Reverse	ATACAAATCCCCAAACTGAATAACA
RD29A	Forward	CACAATCACTTGGCTCCACTGTTG
	Reverse	ACCTAGTAGCTGGTATGGAGGAACT
RD29B	Forward	GTTGAAGAGTCTCCACAATCACTTG
	Reverse	ATACAAATCCCCAAACTGAATAACA
ABI1	Forward	GTTTGGGATGTAATGACGGATG
	Reverse	TGAACTGAGGCAGAGAGGGTCC
ABI2	Forward	AGAAAAGAGGAGAAGGAAAAGATCC
	Reverse	TAAAGAGAATTTTTACCCACCATCA
HAB1	Forward	GACTACCTCTCAATGCTTGCTCTAC
	Reverse	AAAAACCTGTCGAAATTAGATCCTT
AtActin8	Forward	CAACTATGTTCTCAGGTATTGCAGA
	Reverse	GTCATGGAAACGATGTCTCTTTAGT
